# Trends in Hyperinsulinemia and Insulin Resistance Among Nondiabetic US Adults, NHANES, 1999–2018

**DOI:** 10.3390/jcm14093215

**Published:** 2025-05-06

**Authors:** Chuyue Wu, Yixun Ke, Roch A. Nianogo

**Affiliations:** 1 Department of Epidemiology, Fielding School of Public Health, University of California Los Angeles (UCLA), Los Angeles, CA 90095, USA; ke0601@g.ucla.edu (Y.K.); niaroch@ucla.edu (R.A.N.); 2 California Center for Population Research, University of California Los Angeles (UCLA), Los Angeles, CA 90095, USA

**Keywords:** hyperinsulinemia, insulin resistance, diabetes, metabolic disorders, temporal trend, NHANES, sociodemographic disparities

## Abstract

**Introduction:** Hyperinsulinemia and insulin resistance are strong predictors of cardiometabolic diseases, which disproportionately affect individuals across gender, racial/ethnic, and socioeconomic groups. We aim to estimate and test the temporal trends in the prevalence of hyperinsulinemia and insulin resistance (IR) by sociodemographic groups among nondiabetic adults in the United States from 1999 to 2018. **Methods:** We used data from the National Health and Nutrition Examination Survey (NHANES) from 1999 to 2018. We fitted linear and joinpoint logistic regression models to test the sample weighted and age-standardized time trends for linear and nonlinear trends in the prevalence of hyperinsulinemia and IR, respectively. **Results:** We included 17,310 nondiabetic men and nonpregnant women aged 20 years or older. The age-standardized prevalence of hyperinsulinemia increased from 28.2% in 1999–2000 to 41.4% in 2017–2018, with IR prevalence similarly rising from 24.8% in 1999–2000 to 38.4% in 2017–2018. Across the entire period examined, individuals who were male; non-Hispanic Black; Hispanic; or had a lower educational level or lower family income consistently had a higher prevalence of hyperinsulinemia and IR than other groups. We found increasing temporal trends in the prevalence of hyperinsulinemia and IR for all the sociodemographic subgroups, at least in some periods from 1999 to 2018. **Conclusions:** There was an increased age-standardized prevalence of hyperinsulinemia and IR among nondiabetic adults in the US across each defined sociodemographic group from 1999 to 2018. The difference in prevalence across subgroups underscores the need for designing personalized and targeted interventions to address disparities.

## 1. Background

Insulin resistance (IR), characterized by the body tissue’s (including skeletal muscles, liver, and adipose tissue) reduced responsiveness to insulin, is tightly connected to and often coexists with hyperinsulinemia, a condition of excess insulin levels in the bloodstream [1]. Hyperinsulinemia can result from IR when β-cell compensatively produces more insulin to overcome IR and maintain normal blood glucose levels. However, insulin hypersecretion or hyperinsulinemia can also precede the development of IR [2,3,4], and the secondary IR may act as the downstream defense mechanism to reduce the metabolic stress to critical organs and prevent hypoglycemia [5]. Nevertheless, although the causal relationship between hyperinsulinemia and IR remains debated [6], it is widely agreed that both hyperinsulinemia and IR are precursors of type 2 diabetes and are critical underlying components of metabolic syndrome [7,8]. Furthermore, numerous studies have explored the relation of hyperinsulinemia or IR to cardiovascular disease [9,10], cancer [9,11], nonalcoholic fatty liver disease (NAFLD) [12], polycystic ovary syndrome (PCOS) [13], chronic kidney disease (CKD) [14,15], and dementia [16], which indicates the essential role of these two factors in the development of chronic diseases. Since hyperinsulinemia and IR can be detectable long before the onset of overt type 2 diabetes [17] and are modifiable through improving lifestyle behaviors or environmental risk factors [3], studying the temporal trends in these factors is critical to understanding how these conditions are evolving over time, especially in the context of rising chronic disease burden and sociodemographic disparities.

Research has documented the increased prevalence of obesity, metabolic syndrome, type 2 diabetes, and cardiovascular risk factors [18,19,20,21,22]. Racial and ethnic minority populations are disproportionately affected by metabolic disorders, including elevated blood glucose levels and other cardiovascular risk factors [18,22]. The disparities of metabolic disorders and type 2 diabetes exist also across different socioeconomic groups, as individuals from lower socioeconomic backgrounds may adhere to unhealthier lifestyles and be more likely to lack awareness [20,22,23]. With the disproportionally increased prevalence of metabolic disorders by racial/ethnic and socioeconomic groups, it is important to investigate whether there exists an increasing trend in hyperinsulinemia and IR since they represent essential underlying risk factors for diabetes and the metabolic syndrome. Understanding the trends in the prevalence of hyperinsulinemia and IR, particularly the disparities across different sociodemographic groups can inform public health planning, resource allocation, and the development of public health policies to reduce the hyperinsulinemia/IR-related disease burden.

A previous study by Li et al. [24] found that the prevalence of hyperinsulinemia increased by 35.1%, and the mean fasting insulin concentrations increased by 5% among nondiabetic US adults from 1988 to 2002. In the context of increased burden of poor diet, high body mass index (BMI), and high blood glucose levels in the US in the last two decades [25], our study aims to (i) investigate the trend in the prevalence of hyperinsulinemia and IR among nondiabetic adults in the US from 1999 through 2018 and (ii) investigate the presence of disparities in these trends by gender, racial/ethnic, and socioeconomic subgroups in the U.S.

## 2. Method

### 2.1. Study Design and Study Population

We used the serial cross-sectional National Health and Nutrition Examination Survey (NHANES) from 1999 to 2018. NHANES applied a multistage, clustered probability sampling design, making it representative of the US civilian noninstitutionalized population [26].

**Figure 1:**
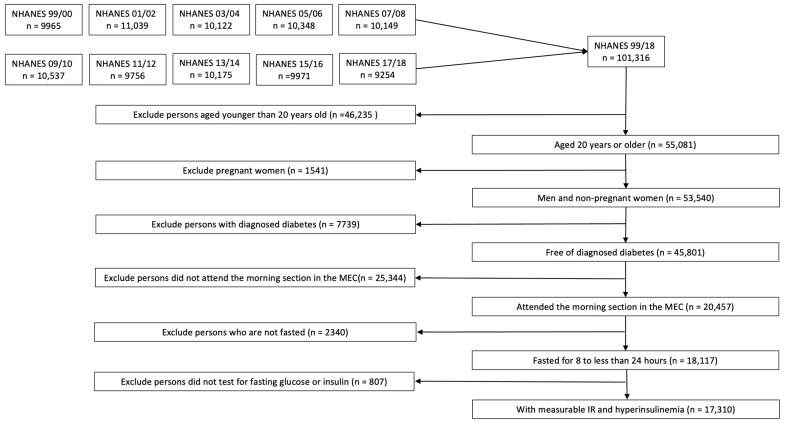
Study population flow diagram. Figure legend: flowchart illustrating the inclusion and exclusion criteria applied to the NHANES 1999–2018 dataset, resulting in a final sample of 17,310 adult participants as the study population of interest. Abbreviations: NHANES, National Health and Nutrition Examination Survey; MEC, Mobile Examination Center.

We defined our population of interest as follows. We included participants who (i) were male and nonpregnant female adults aged 20 years or older, (ii) were free of diabetes, (iii) attended the morning Mobile Examination Center (MEC) session, and (iv) had fasted for 8 to 24 h. Diabetes was defined as (i) an affirmative answer to the question “Has a doctor ever told having diabetes?” or (ii) having a fasting glucose level equal to or greater than 126 mg/dl. Pregnancy status was determined based on self-report or a positive urine pregnancy test. Out of 101,316 participants across 10 continuous NHANES cycles, we excluded individuals under 20 years (*n* = 46,235), pregnant women (*n* = 1541), those with diabetes (*n* = 7739), non-morning session attendees (*n* = 25,344), non-fasters (*n* = 2340), and individuals not tested for glucose or insulin (*n* = 807), resulting in a final analytical study sample of 17,310 participants (Figure 1). Among these, 19 had missing data for educational level, and 1538 had missing data for the poverty–income ratio (PIR). The analyses related to non-Hispanic Asians had 7066 participants starting from 2011 to 2018.

### 2.2. Data Collection

We identified sex, race/ethnicity, education, and income level as potential effect measure modifiers in the trends of hyperinsulinemia and IR. Self-reported race/ethnicity included non-Hispanic White, non-Hispanic Black, Hispanic, non-Hispanic Asian, and other. Non-Hispanic Asians identified as a subracial group since the 2011–2012 cycle. The highest level of education was classified as high school or less, some college or associate’s degree, and college graduate or higher. We used the poverty–income ratio (PIR) as an income indicator, which measures family income relative to the federal poverty level (FGL). The PIR index was calculated by dividing family’s income by the poverty threshold for their family size and survey year in NHANES, which was comparable over time with the adjustment of inflation [27]. We further stratified the continuous PIR index into three categories: equal or lower than 1.3 (low income), greater than 1.3 to 3.5 (middle income), and above 3.5 (high income) [28]. The demographic questionnaire information was collected in the home interviews by trained interviewers using the computer-assisted personal interviewing (CAPI) system, with subsequent review by NHANES staff to ensure accuracy and completeness.

The assay and lab site for measuring fasting insulin concentration varied cycle by cycle. Therefore, we used the Tosoh analyzer immunoenzymometric method as the standard method and adjusted the insulin measures from other cycles based on the regression equations provided by the NHANES website [29,30,31,32], ensuring comparability across cycles (Supplementary Table S1). Hyperinsulinemia was defined as a fasting insulin level greater than 10 µU/mL, which was consistent with a previous study [24] and was greater than the median values across each cycle. Insulin resistance was quantified using the Homeostatic Model Assessment of Insulin Resistance (HOMA-IR) [33], a validated method strongly correlated with the gold-standard hyperinsulinemic euglycemic clamp (HEC) method for measuring IR [34,35,36,37]. The HOMA-IR index was calculated using the formula [33]:

$${HOMA}_{IR\;index}=fasting\;insulin\left[µU/mL\right]\times fasting\;glucose[mmol/L]/22.5.$$

We chose the 66.7 percentile, which is 2.6, of the HOMA-IR index as the cutoff point, identifying the upper third of the surveyed population as insulin-resistant, as previously conducted in a study using NHANES 1999–2002 [38].

To analyze the nationally representative insulin levels, we applied designated fasting subsample weights (WTSAF2YR) that account for the probability of selection and nonresponse in the subsample [39,40,41,42]. Additionally, we directly standardized our study population to the 2010 U.S. census population [43] to account for the change in age distribution over time. Final weights were derived by multiplying fasting subsample weights by age standardization weights.

### 2.3. Statistical Analysis

We describe the study participants’ sociodemographic profiles across each two-year cycle. We reported weighted means with standard deviations or medians with interquartile ranges for continuous variables while we provided unweighted frequencies and corresponding weighted percentages for categorical variables.

To assess trend nonlinearity, we employed a stepwise approach [44]. We performed polynomial regression analyses, starting with a cubic term for the time variable. A lack of statistical significance in the cubic term led to model simplification by considering only the quadratic term. A linear trend was finally presumed if the quadratic term was also insignificant. Since data among the non-Hispanic Asian subgroup were only available from 2011 to 2018, we assumed this subgroup’s trends were linear without assessing nonlinearity. Subsequent trend analyses proceeded with logistic regression for the binary outcomes.

In cases where either cubic or quadratic terms were significant, which indicated nonlinearity, we employed the National Cancer Institute’s (NCI’s) Joinpoint Regression Program (version 5.0.2) [45] to pinpoint joinpoints, i.e., the points of inflection where shifts in the trend occurred. We constrained our analysis to detect one joinpoint consistent with the recommended settings for datasets with 10 time points [44]. For nonlinear trend analysis, we fitted the joinpoint regression model, which consists of two linear segments that have different slopes intersecting at the joinpoint [46]. However, if adjacent linear segments did not differ significantly in slope, we removed the joinpoint and refitted the model as linear. This refinement ensured that only statistically significant changes in the trend were modeled by fitting joinpoint regression [44].

We conducted two sets of analyses for each outcome: age-adjusted and fully adjusted. We fitted the fully adjusted models by adjusting for age, sex, race/ethnicity, education level, and PIR due to their potential role as confounders of the trends in hyperinsulinemia and IR. We also added the interaction terms between time and each covariate to test the possible effect measure modification of the trends across each sociodemographic subgroup.

We conducted additional sensitivity analyses using the same method to test the trends in fasting insulin and HOMA-IR index as continuous outcome variables. Since the distribution of fasting insulin and HOMA-IR index were right-skewed, we log-transformed these two variables to normalize the distributions and calculated the means for each survey cycle.

We performed all statistical analyses using SAS software (version 9.4, SAS Institute Inc., Cary, NC, USA) with the SURVEY procedures, which accounted for the complex survey design of NHANES, incorporating weights, strata, and primary sampling units to ensure national representativeness. We used R programing (version 4.4.0) for figure plotting.

## 3. Results

### 3.1. Participant Characteristics

On average, about 45% were aged 20–39, 42% were 40–59, and 13% were 60 or older, with a slight upward trend in mean age over cycles, starting from 41.3 ± 0.6 years to 43.0 ± 0.6 years. The proportion of women remained stable at around 51–52%. The sample was predominantly composed of Non-Hispanic White individuals (68%), followed by Hispanic (14%) and Non-Hispanic Black individuals (11%), reflecting the general U.S. nondiabetic adult population. Non-Hispanic Asian individuals were oversampled and recorded after 2011 with a proportion of about 6%. Educational level showed improvement over the study period, with college graduates increasing from 25% to 31% and those with high school education or less decreasing from 48% to 38%. Regarding poverty level, on average, 20% had a PIR below 1.3, 36% between 1.3 and less than 3.5, and 44% above 3.5, indicating a socioeconomically diverse sample (Table 1).

### 3.2. Overall Trends in the Prevalence of Hyperinsulinemia and IR

From 1999 to 2018, there was a significant increase in the age-standardized prevalence of both hyperinsulinemia and IR across the total population. The prevalence of hyperinsulinemia rose from 28.2% (95% CI: 24.2–32.1%) to 41.4% (95% CI: 37.4–45.5%), while IR increased from 24.8% (95% CI: 21.6–28.0%) to 38.4% (95% CI: 34.4–42.4%). This growth was most pronounced from 1999 to 2010 (*p* for trend <0.0001), with rates stabilizing between 2010 and 2018.

### 3.3. Trends in the Prevalence of Hyperinsulinemia and IR by Gender

Males showed a relatively higher age-standardized prevalence of hyperinsulinemia than females from 1999 to 2018 (male: 31.8% [95%CI: 26.4–37.2%] to 41.7% [95%CI: 34.9–48.5%]; females: 24.7% [95%CI: 20.8–28.6%] to 41.2% [95%CI: 33.0–49.5%]). We found females had an increasing trend in the prevalence of hyperinsulinemia and IR from 1999 to 2018 (*p* for trend <0.0001), while this prevalence in males remained stable after 2010, with the differences across sexes diminishing from 2010 to 2018 as a result.

### 3.4. Trends in the Prevalence of Hyperinsulinemia and IR by Race/Ethnicity

All racial/ethnic subgroups experienced an increase in the prevalence of hyperinsulinemia and IR, with Hispanic and Non-Hispanic Black individuals consistently showing higher rates compared to Non-Hispanic Whites. For Non-Hispanic Whites and Blacks, the prevalence increased from 1999 to 2010, after which it stabilized for Whites and decreased for Blacks. Non-Hispanic Asians showed a marked increase in prevalence from 2011 onwards, compared to the rates of Non-Hispanic Whites.

### 3.5. Trends in the Prevalence of Hyperinsulinemia and IR by Socioeconomic Status

**Figure 2:**
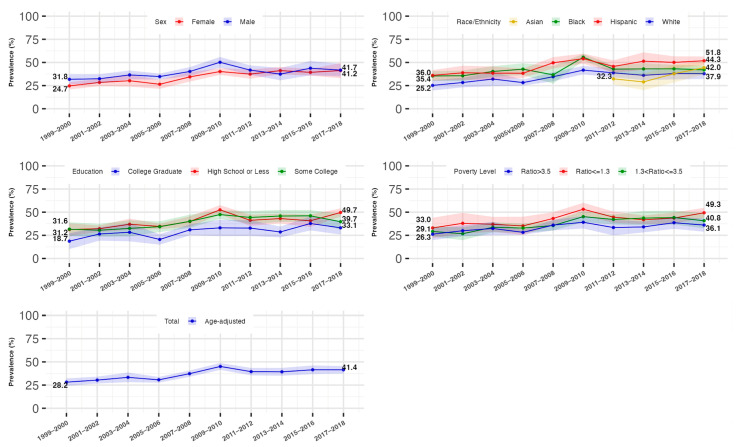
Age-standardized prevalence and 95% confidence interval of hyperinsulinemia stratified by sex, race/ethnicity, and educational and income level, National Health and Nutrition Examination Survey (NHANES), 1999 to 2018. Figure legend: showing the trends in the age-standardized prevalence of hyperinsulinemia and the disparities across sociodemographic groups. Hyperinsulinemia was defined as fasting insulin levels greater than 10 µU/mL. The prevalence of hyperinsulinemia and the corresponding 95% confidence interval were survey-sample-weighted and age-standardized to 2010 U.S. Census adult population. The sample size for the total, sex-stratified, and race/ethnicity (without Asian)-stratified trends was 17,310. The sample size for the stratified non-Hispanic Asian subgroup was 7066. A total of 19 participants were excluded from the education-stratified trend analyses. A total of 1538 participants were excluded from the income-stratified trend analyses.

**Figure 3:**
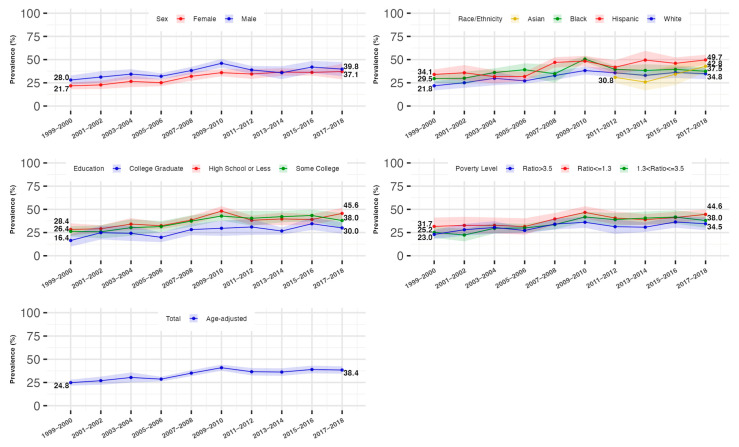
Age-standardized prevalence and 95% confidence interval of insulin resistance stratified by sex, race/ethnicity, and educational and income level, National Health and Nutrition Examination Survey (NHANES), 1999 to 2018. Figure legend: showing the trends in the age-standardized prevalence of insulin resistance and the disparities across sociodemographic groups. Insulin resistance was defined as a Homeostatic Model Assessment of Insulin Resistance (HOMA-IR) index greater than 2.6 (the 66.7th percentile). The prevalence of insulin resistance and the corresponding 95% confidence interval were survey-sample-weighted and age-standardized to 2010 U.S. Census adult population. The sample size for the total, sex-stratified, and race/ethnicity (without Asian)-stratified trends was 17,310. The sample size for the stratified non-Hispanic Asian subgroup was 7066. A total of 19 participants were excluded from the education-stratified trend analyses. A total of 1538 participants were excluded from the income-stratified trend analyses.

The age-standardized prevalence rose similarly across all education and family income levels, with the lowest-educated (high school or less) and lowest-family-income (PIR ≤ 1.3) individuals consistently experiencing a higher prevalence of hyperinsulinemia, compared to the highest-educated (college graduate) and highest-family-income (PIR > 3.5) individuals, respectively. Fully adjusted analyses confirmed similar trends across all sociodemographic groups from 1999 to 2018. These trends are shown in Table 2 and Table 3 and Figure 2 and Figure 3.

Additional analyses revealed consistent trends and disparities in continuous log-transformed fasting insulin levels and HOMA-IR index across all sociodemographic subgroups (Supplementary Section, Tables S2 and S3, Figures S1 and S2).

## 4. Discussion

This study analyzed the temporal trends of hyperinsulinemia and insulin resistance (IR) among nondiabetic U.S. adults from 1999 to 2018, revealing a significant and widespread increase in both conditions. Our study showed that males consistently had higher insulin levels and a greater prevalence of hyperinsulinemia and IR compared to females, although females exhibited a more rapid increase in these conditions over the last four cycles. Notably, non-Hispanic Asians displayed a faster growth rate in these conditions compared to other racial/ethnic groups since 2011. Additionally, non-Hispanic Black and Hispanic individuals consistently showed higher prevalence rates of hyperinsulinemia and IR, as well as higher levels of fasting insulin and HOMA-IR index, compared to non-Hispanic White individuals. Those with lower educational and income levels were also more likely to have higher insulin levels and a greater prevalence of hyperinsulinemia and IR. These findings highlight the need for targeted interventions to address these disparities, especially among racial/ethnic minority groups and those of lower socioeconomic status, to reduce health inequalities and mitigate the risks associated with chronic diseases.

Our findings extend the results of earlier research by Li et al. [24] showing that the overall age-standardized log-scale fasting insulin levels increased by 8.6% from 2002 to 2018, doubling the 4.9% increase reported by Li et al. from 1988 to 2002. Hyperinsulinemia prevalence escalated by 46.8% overall from 1999 to 2018. The growth rate was notably higher in women (66.8%) compared to men (31.1%), contrasting with the more balanced increase rates reported by Li et al. (male vs. female: 38.3% vs. 32.1%). The trends in hyperinsulinemia and IR observed in our study also align with the rising prevalence of obesity, metabolic syndrome, type 2 diabetes, and cardiovascular risk factors reported by previous studies [18,19,20,21,22]. For instance, the prevalence of metabolic syndrome significantly increased among women and non-Hispanic Asians from 2011 to 2016 [19], while a higher prevalence of type 2 diabetes was observed among adult men and minority racial/ethnic groups [20]. These parallel trends underscore the potential causal relationship between hyperinsulinemia/IR and future severe metabolic disorders, reinforcing the need for targeted interventions to reduce these risks.

The observed increase in hyperinsulinemia and IR over time can be attributed to rising trends in traditional risk factors, such as high-calorie diets and physical inactivity [47]. However, other factors may also contribute to these trends. Dr. Corkey proposed that environmental risk factors, combined with a genetic predisposition, could lead to elevated basal insulin levels, which, in turn, might be the root cause of insulin resistance, obesity, and type 2 diabetes [3]. Environmental changes, including the presence of toxins like polybrominated diphenyl ethers and the introduction of new substances in the food supply (e.g., artificial sweeteners, preservatives, emulsifiers, and flavor enhancers), could potentially drive insulin hypersecretion, leading to future metabolic diseases. This suggests that future research should shift its focus from traditional risk factors alone to exploring the impact of environmental agents on insulin secretion and metabolic health.

The gender differences observed in this study, where males exhibited a higher prevalence of hyperinsulinemia and IR, as well as higher fasting insulin levels and HOMA-IR index compared to females, may result from a complex interplay of biological and societal factors [48,49]. Biologically, women benefit from the insulin-sensitizing effects of estrogen until menopause, whereas men tend to have higher levels of androgens and more visceral fat, both linked to increased insulin resistance. Additionally, differences in lifestyle behaviors, such as dietary preferences and physical activity levels, likely contribute to these disparities. The continuous increase in hyperinsulinemia and IR among women, compared to the stable trends in men, warrants further investigation into potential risk factors that disproportionately affect women, such as environmental toxins, stress, antidepressant use, and sedentary behavior. Identifying and addressing these factors is crucial to reducing health disparities and improving the management of hyperinsulinemia and IR.

Our study also found that non-Hispanic Blacks and Hispanics consistently had higher age-standardized levels of fasting insulin, HOMA-IR index, and greater prevalence of hyperinsulinemia and IR compared to non-Hispanic Whites. This finding aligns with previous research and may be attributed to a combination of biological and sociocultural factors [50,51]. For example, African Americans tend to have more robust beta-cell function than non-Hispanic Whites, characterized by primary insulin hypersecretion, which presents a unique risk profile. This calls for further research to determine whether intensive lifestyle modification strategies and/or medications that enhance beta-cell function, such as GLP-1 agonists and thiazolidinediones, are suitable and effective for managing type 2 diabetes in African Americans [51]. Additionally, sociocultural factors, such as limited access to healthy foods [52], less walkable communities, lower health awareness, and higher levels of chronic stress, may contribute to the higher prevalence of hyperinsulinemia and IR among lower-educated and lower-income populations. Previous studies have suggested that childhood adversity and adult stress, mediated by inflammatory and hypothalamic–pituitary–adrenal (HPA) axis responses, significantly influence insulin resistance and may explain the disparities observed across socioeconomic statuses [53]. Addressing these sociodemographic factors is essential for reducing disparities in the development of hyperinsulinemia, IR, and related metabolic diseases. Future research should focus on designing personalized management strategies for individuals from different sociodemographic groups.

Our study is not without limitations. The decreased response rate for MEC examinations (which decreased from 80% in 2001 to 49% in 2018) could have introduced selection bias. Still, this missingness is likely random, and an investigation of nonresponse bias in 2017–2018 NHANES conducted by Fakhouri THI et al. found that the increasing nonresponse rate had little effect on the final weighted estimates [54], ensuring comparability with previous NHANES cycles. Additionally, the evolution of type 2 diabetes definitions over time, particularly the ADA’s 2009 inclusion of the HbA1c test for diagnosis [55], could have led to the inclusion of diabetic individuals in pre-2009 cycles, potentially overestimating insulin levels in pre-2009 cycles. Nonetheless, these definitional differences would likely result in more conservative trend estimates than the actual effects. Variability in insulin assay methods across survey cycles, despite efforts to standardize them, may also result in nondifferential measurement errors. Furthermore, the lack of consensus on defining cutoff points for hyperinsulinemia and HOMA-IR across different sociodemographic groups may affect the generalizability and reproducibility of the findings. Further studies are needed to establish sex- and racial/ethnical-specific cutoff points that are more predictive for future relevant outcome risks [36].

## 5. Conclusions

We found a widespread rise in hyperinsulinemia and IR prevalence and fasting insulin concentrations among nondiabetic adults in the US from 1999 to 2018. We observed disparities where male, non-Hispanic Black, Hispanic, and lower socioeconomic individuals experienced a higher prevalence of hyperinsulinemia and IR and noted that females and non-Hispanic Asians showed a more alarming increasing rate of hyperinsulinemia and IR in recent years from 2011 to 2018. These findings underscore the need for early detection and treatment to prevent the progression of hyperinsulinemia and IR among nondiabetic individuals and the importance of addressing disparities across sociodemographic subgroups.

## Figures and Tables

**Table 1:** Overall characteristics of study population, National Health and Nutrition Examination Survey (NHANES), 1999 to 2018, *n* = 17,310.

Characteristics	Overall *n* = 17,310	1999–2000 *n* = 1461	2001–2002 *n* = 1695	2003–2004 *n* = 1567	2005–2006 *n* = 1521	2007–2008 *n* = 1875	2009–2010 *n* = 2125	2011–2012 *n* = 1768	2013–2014 *n* = 1901	2015–2016 *n* = 1710	2017–2018 *n* = 1687
Age distribution, *n* * (%) ^†^, years											
20–39	6381 (41)	494 (45)	623 (44)	552 (42)	576 (40)	671 (41)	774 (40)	726 (39)	701 (41)	647 (41)	617 (40)
40–59	5782 (38)	455 (35)	567 (39)	475 (38)	506 (40)	625 (39)	758 (39)	578 (38)	684 (37)	577 (35)	557 (35)
≥60	5147 (21)	512 (20)	505 (17)	540 (19)	439 (20)	579 (20)	593 (21)	464 (23)	516 (23)	486 (24)	513 (24)
**Age, Mean (SD) ^†^, years**	45.3 (0.2)	44.3 (0.7)	44.0 (1.0)	44.8 (0.6)	45.5 (0.8)	45.2 (0.6)	45.4 (0.7)	46.0 (0.7)	45.6 (0.7)	45.6 (0.7)	46.3 (0.6)
Female sex, *n* * (%) ^†^	8890 (51)	737 (51)	843 (51)	783 (51)	709 (49)	972 (51)	1149 (51)	890 (52)	996 (51)	903 (52)	908 (52)
Race and Ethnicity, *n* * (%) ^†^											
Non-Hispanic White	7987 (68)	679 (71)	944 (73)	866 (71)	792 (72)	924 (70)	1035 (67)	712 (67)	860 (65)	599 (62)	576 (61)
Non-Hispanic Black	3241 (11)	251 (10)	281 (10)	287 (11)	332 (11)	327 (10)	344 (11)	367 (11)	338 (12)	336 (12)	378 (11)
Hispanic	4444 (14)	496 (16)	420 (12)	345 (12)	328 (11)	545 (14)	630 (14)	369 (15)	421 (16)	500 (16)	390 (17)
Non-Hispanic Asian ^‡^	976 (6)	NA	NA	NA	NA	NA	NA	277 (6)	237 (5)	216 (6)	246 (6)
Other ^§^ (Including Asian)	1638 (7)	35 (4)	50 (4)	69 (6)	69 (6)	79 (6)	116 (7)	320 (8)	282 (8)	275 (11)	343 (11)
Other ^§^ (Excluding Asian)	244 (3)	NA	NA	NA	NA	NA	NA	43 (2)	45 (2)	59 (4)	97 (5)
Educational Level, *n* * (%) ^†^											
High school or less	8272 (39)	857 (48)	860 (42)	815 (42)	736 (40)	961 (40)	1053 (39)	731 (34)	790 (35)	742 (36)	727 (38)
Some college or associate’s degree	5005 (32)	344 (27)	489 (34)	437 (33)	470 (33)	491 (30)	604 (30)	542 (33)	583 (32)	504 (32)	541 (32)
College graduate or higher	4014 (29)	257 (25)	343 (24)	313 (25)	312 (27)	422 (30)	464 (30)	495 (33)	527 (32)	464 (32)	417 (31)
Frequency Missing	19	3	3	2	3	1	4	0	1	0	2
Poverty–Income Ratio, *n* * (%) ^†^											
Ratio ≤ 1.3	4538 (20)	326 (18)	360 (18)	392 (20)	343 (14)	491 (19)	637 (22)	536 (24)	596 (25)	460 (21)	397 (20)
1.3 < Ratio ≤ 3.5	6051 (36)	486 (34)	618 (35)	592 (36)	590 (38)	661 (33)	712 (37)	566 (34)	593 (33)	619 (38)	614 (37)
Ratio > 3.5	5183 (44)	450 (47)	599 (46)	481 (44)	521 (48)	570 (49)	572 (41)	516 (42)	570 (41)	453 (41)	451 (43)
Frequency Missing	1538	199	118	102	67	153	204	150	142	178	225
**Fasting glucose, Mean (SD) ^†^, mmol/L**	5.4 (0.01)	5.2 (0.03)	5.3 (0.02)	5.3 (0.03)	5.4 (0.03)	5.5 (0.02)	5.4 (0.02)	5.4 (0.02)	5.4 (0.03)	5.5 (0.02)	5.6 (0.02)
Fasting insulin **, Median (q1, q3) ^†^, μU/mL	7.8 (4.7, 13.0)	6.8 (4.2, 10.9)	6.7 (4.2, 11.2)	7.3 (4.4, 12.2)	6.5 (3.6, 11.9)	7.8 (4.3, 13.0)	9.0 (5.2, 15.4)	8.2 (5.3, 13.7)	8.2 (5.2, 13.2)	8.4 (5.4, 14.0)	8.5 (5.6, 13.6)
**HOMA-IR, Median (q1, q3) ^†^**	1.9 (1.1, 3.2)	1.6 (0.9, 2.6)	1.6 (1.0, 2.7)	1.7 (1.0, 3.0)	1.5 (0.8, 3.0)	1.9 (1.0, 3.2)	2.1 (1.2, 3.8)	2.0 (1.2, 3.4)	2.0 (1.2, 3.2)	2.1 (1.3, 3.5)	2.1 (1.4, 3.5)

Abbreviations: SD, standard deviation; Poverty–Income Ratio, i.e., PIR, defined as family income divided by the federal poverty level (FGL); HOMA-IR, Homeostatic Model Assessment of Insulin Resistance, calculated by fasting insulin level in μU/mL times fasting glucose level in mmol/l divided by 22.5; NA, not applicable. * *n* was the unweighted sample size. The total number of participants was 17,310. A total of 19 (0.1%) participants had a missing value of educational level and 1538 (8.9%) participants had a missing value of poverty–income ratio. **^†^** %, mean (SD) and median (q1, q3) were survey-sample weighted and age-standardized (age distribution and continuous age were only sample weighted but not age-standardized). We used 2010 U.S. Census adult population as the standard population; the age groups were: 20- < 25 (9.6%); 25- < 30 (9.8%); 30- < 35 (8.9%); 35- < 40 (8.9%); 40- < 45 (9.3%); 45- < 50 (10.2%); 50- < 55 (9.9%); 55- < 60 (8.7%); 60- < 65 (7.3%); 65- < 70 (5.5%); 70- < 75 (4.0%); 75- < 80 (3.3%); 80- < 85 (2.6%); and ≥85 (2.1%). ^‡^ Representative information for non-Hispanic Asian population was available in the NHANES only from 2011 through 2018. The sample size was 7066 (i.e., the study population from last for survey cycles, 2011 to 2018). ^§^ Other race/ethnicity includes Native American/Alaskan, multiracial, and all other responses. ** fasting insulin concentration is standardized using the Tosoh analyzer immunoenzymometric method.

**Table 2:** Test for trend in the prevalence of hyperinsulinemia stratified by sex, race/ethnicity, and educational and income level, National Health and Nutrition Examination Survey (NHANES), 1999 to 2018, *n* = 17,310.

	Age-Adjusted Models				
	Joinpoint Wave ^†^	OR (95%CI) *p*-Value		Contrast *p*-Value ^‡^	Interaction *p*-Value ^§^
		Segment 1	Segment 2		
**Overall**	6	1.13 (1.09–1.17) <0.001	1.00 (0.96–1.04) 0.99	<0.001	NA
**Sex**					
Female	NA	1.09 (1.06–1.12) <0.001		NA	0.062
Male	6	1.13 (1.08–1.19) <0.001	0.96 (0.90–1.03) 0.25	0.002	ref
**Race/Ethnicity ^||^**					
Non-Hispanic White	6	1.14 (1.08–1.19) <0.001	0.99 (0.93–1.05) 0.66	0.004	ref
Non-Hispanic Black	6	1.12 (1.07–1.18) <0.001	0.93 (0.88–0.99) 0.027	<0.001	0.098
Hispanic	NA	1.08 (1.05–1.11) <0.001		NA	0.67
Non-Hispanic Asian ^¶^	NA	1.22 (1.08–1.38) 0.003		NA	0.005
Educational level **					
High school or less	6	1.14 (1.08–1.20) <0.001	1.00 (0.95–1.06) 1.00	0.007	0.93
Some college or associate’s degree	7	1.14 (1.08–1.20) <0.001	0.94 (0.84–1.04) 0.22	0.006	0.98
College graduate or higher	NA	1.08 (1.03–1.12) <0.001		NA	ref
**Poverty–Income Ratio ^††^**					
Ratio ≤ 1.3	NA	1.06 (1.02–1.10) 0.002		NA	0.66
1.3 < Ratio ≤ 3.5	8	1.12 (1.08–1.17) <0.001	0.91 (0.77–1.08) 0.29	0.034	0.087
Ratio > 3.5	NA	1.05 (1.02–1.08) 0.002		NA	ref
	**Fully Adjusted ^‡‡^ Models**				
	**Joinpoint Wave ^†^**	**OR (95%CI)** ***p*-Value**		**Contrast *p*-Value ^‡^**	**Interaction *p*-Value ^§^**
		Segment 1	Segment 2		
**Overall**	6	1.13 (1.09–1.18) <0.001	1.00 (0.95–1.04) 0.84	<0.001	NA
**Sex**					
Female	NA	1.09 (1.06–1.12) <0.001		NA	0.033
Male	6	1.13 (1.08–1.19) <0.001	0.96 (0.90–1.02) 0.191	0.001	ref
**Race/Ethnicity ^||^**					
Non-Hispanic White	6	1.15 (1.09–1.21) <0.001	0.98 (0.93–1.04) 0.58	0.002	ref
Non-Hispanic Black	6	1.11 (1.05–1.17) <0.001	0.93 (0.87–1.00) 0.040	0.002	0.023
Hispanic	NA	1.08 (1.05–1.12) <0.001		NA	0.52
Non-Hispanic Asian ^¶^	NA	1.20 (1.03–1.40) 0.019		NA	0.022
Educational level **					
High school or less	6	1.13 (1.07–1.20) <0.001	0.99 (0.93–1.05) 0.65	0.008	0.92
Some college or associate’s degree	7	1.13 (1.07–1.20) <0.001	0.95 (0.84–1.06) 0.33	0.018	0.80
College graduate or higher	NA	1.07 (1.02–1.11) 0.003		NA	Ref
**Poverty–Income Ratio ^††^**					
Ratio ≤ 1.3	NA	1.06 (1.02–1.10) 0.002		NA	0.86
1.3 < Ratio ≤ 3.5	8	0.89 (0.85–0.92) <0.001	1.11 (0.94–1.32) 0.23	0.023	0.113
Ratio > 3.5	NA	1.06 (1.03–1.10) <0.001		NA	ref

Abbreviations: OR, odds ratio; CI, confidence interval; ref, reference; NA, not applicable. * Hyperinsulinemia was defined as fasting insulin levels greater than 10 µU/mL. ^†^ Nonlinearity was assessed by testing for the statistical significance of the cubic term and quadratic term of survey cycles in the polynomial logistic regression models. The locations of joinpoint waves were identified by using the NCI’s Joinpoint software (version 5.0.2) for the nonlinear trends. ^‡^ Contrast p-value tested for the statistical significance of the difference between two segments. ^§^ Interaction p-value tested for the statistical significance of the interaction term between the potential modifiers and survey cycle. ^||^ In the race/ethnicity subgroup analyses, race/ethnicity was categorized as non-Hispanic White, non-Hispanic Black, Hispanic, and Other. All 10 survey cycles are included, with sample size 17,310. ^¶^ In the non-Hispanic Asian subgroup analyses, since representative information for non-Hispanic Asian Americans was available in the NHANES only from 2011 through 2018, the analytic sample size was 7066 (i.e., the study population from the last survey cycles, 2011 to 2018). ** Educational level had 19 missing values, which were excluded from the analyses related to educational level. ^††^ Poverty–income ratio had 1538 missing values, which were excluded from the analyses related to poverty–income ratio. ^‡‡^ The fully adjusted models were adjusted for age, sex, race/ethnicity (non-Hispanic White, non-Hispanic Black, Hispanic, and Other), educational level, and poverty–income ratio. In the subgroup analyses, the stratified variable was eliminated from the fully adjusted models, correspondingly.

**Table 3:** Test for trend in the prevalence of insulin resistance stratified by sex, race/ethnicity, and educational and income level, National Health and Nutrition Examination Survey (NHANES), 1999 to 2018, *n* = 17,310.

	Age-Adjusted Models				
	Joinpoint Wave ^†^	OR (95%CI) *p*-Value		Contrast *p*-Value ^‡^	Interaction *p*-Value ^§^
		Segment 1	Segment 2		
**Overall**	6	1.14 (1.10–1.18) <0.001	1.00 (0.96–1.04) 0.96	<0.001	NA
**Sex**					
Female	NA	1.09 (1.06–1.12) <0.001		NA	0. 101
Male	6	1.13 (1.08–1.18) <0.001	0.98 (0.91–1.04) 0.46	0.004	ref
**Race/Ethnicity ^||^**					
Non-Hispanic White	6	1.15 (1.09–1.21) <0.001	0.98 (0.92–1.04) 0.53	0.002	ref
Non-Hispanic Black	6	1.15 (1.10–1.21) <0.001	0.92 (0.87–0.98) 0.006	<0.001	0.140
Hispanic	NA	1.08 (1.05–1.12) <0.001		NA	0.55
Non-Hispanic Asian ^¶^	NA	1.22 (1.08–1.38) 0.002		NA	0.005
Educational level **					
High school or less	NA	1.08 (1.05–1.11) <0.001		NA	0.93
Some college or associate’s degree	7	1.15 (1.09–1.21) <0.001	0.95 (0.86–1.06) 0.39	0.0116	0.63
College graduate or higher	NA	1.08 (1.04–1.12) <0.001		NA	ref
**Poverty–Income Ratio ^††^**					
Ratio ≤ 1.3	NA	1.06 (1.03–1.10) <0.001		NA	0.65
1.3 < Ratio ≤ 3.5	7	1.16 (1.10–1.22) <0.001	0.97 (0.87–1.08) 0.55	0.0119	0.03
Ratio > 3.5	NA	1.05 (1.02–1.08) 0.001		NA	ref
	**Fully Adjusted ^‡‡^ Models**				
	**Joinpoint Wave ^†^**	**OR (95%CI)** ***p*-Value**		**Contrast *p*-Value ^‡^**	**Interaction *p*-Value ^§^**
		Segment 1	Segment 2		
Overall	6	1.14 (1.10–1.18) <0.001	1.00 (0.96–1.04) 1.00	<0.001	NA
**Sex**					
Female	NA	1.09 (1.06–1.13) <0.001		NA	0.061
Male	6	1.13 (1.08–1.19) <0.001	0.97 (0.91–1.04) 0.41	0.003	ref
**Race/Ethnicity ^||^**					
Non-Hispanic White	6	1.16 (1.10–1.22) <0.001	0.99 (0.93–1.05) 0.62	0.001	ref
Non-Hispanic Black	6	1.13 (1.07–1.19) <0.001	0.92 (0.86–0.98) 0.010	<0.001	0.026
Hispanic	NA	1.09 (1.06–1.12) <0.001		NA	0.52
Non-Hispanic Asian ^¶^	NA	1.20 (1.03–1.39) 0.017		NA	0.030
Educational level **					
High school or less	NA	1.07 (1.04–1.10) <0.001		NA	0.97
Some college or associate’s degree	7	1.14 (1.08–1.21) <0.001	0.96 (0.86–1.08) 0.53	0.030	0.58
College graduate or higher	NA	1.07 (1.03–1.11) <0.001		NA	ref
**Poverty–Income Ratio ^††^**					
Ratio ≤ 1.3	NA	1.06 (1.03–1.10) <0.001		NA	0.87
1.3 < Ratio ≤ 3.5	7	1.17 (1.11–1.23) <0.001	0.96 (0.86–1.07) 0.44	0.007	0.050
Ratio > 3.5	NA	1.07 (1.03–1.10) <0.001		NA	ref

Abbreviations: OR, odds ratio; CI, confidence interval; ref, reference; NA, not applicable. * Insulin resistance was defined as a Homeostatic Model Assessment of Insulin Resistance (HOMA-IR) index greater than 2.6 (the 66.7th percentile). ^†^ Nonlinearity was assessed by testing for the statistical significance of the cubic term and quadratic term of survey cycles in the polynomial logistic regression models. The locations of joinpoint waves were identified by using the NCI’s Joinpoint software (version 5.0.2) for the nonlinear trends. ^‡^ Contrast *p*-value tested for the statistical significance of the difference between two segments. ^§^ Interaction *p*-value tested for the statistical significance of the interaction term between the potential modifiers and survey cycle. ^||^ In the race/ethnicity subgroup analyses, race/ethnicity was categorized as non-Hispanic White, non-Hispanic Black, Hispanic, and Other. All 10 survey cycles are included, with sample size 17,310. ^¶^ In the non-Hispanic Asian subgroup analyses, since representative information for non-Hispanic Asian population was available in the NHANES only from 2011 through 2018, the analytic sample size was 7066 (i.e., the study population from the last survey cycles, 2011 to 2018). ** Educational level had 19 missing values, which were excluded from the analyses related to educational level. ^††^ Poverty–income ratio had 1538 missing values, which were excluded from the analyses related to poverty–income ratio. ^‡‡^ The fully adjusted models were adjusted for age, sex, race/ethnicity (non-Hispanic White, non-Hispanic Black, Hispanic, and Other), educational level, and poverty–income ratio. In the subgroup analyses, the stratified variable was eliminated from the fully adjusted models, correspondingly.

## Data Availability

A version of the NHANES dataset can be obtained freely from the U.S. Centers for Disease Control and Prevention (CDC) website at https://www.cdc.gov/nchs/nhanes/index.html (accessed on 28 January 2025).

## Supplementary Materials

The following supporting information can be downloaded at: https://www.mdpi.com/article/10.3390/jcm14093215/s1, Section: Trends in the means of natural log-scale fasting insulin levels and natural log-scale HOMA-IR index levels by sociodemographic subgroups. Table S1: The Change of Insulin Measurement Method and Recommended Adjustment Formula from the NHANES Website. Table S2: Test for Trend in the Means of Log-scale Fasting Insulin Levels Stratified by Sex, Race/Ethnicity, Educational and Income level, National Health and Nutrition Examination Survey (NHANES), 1999 to 2018, n = 17,310. Table S3: Test for Trend in the Means of Log-scale HOMA-IR Index Stratified by Sex, Race/Ethnicity, Educational and Income level, National Health and Nutrition Examination Survey (NHANES), 1999 to 2018, n = 17,310. Figure S1: Age-standardized Mean and 95%CI of Natural Log-scale Fasting Insulin Levels Stratified by Sex, Race/Ethnicity, Educational and Income level, National Health and Nutrition Examination Survey (NHANES), 1999 to 2018. Figure S2. Age-standardized Mean and 95%CI of Natural Log-scale HOMA-IR Index Stratified by Sex, Race/Ethnicity, Educational and Income level, National Health and Nutrition Examination Survey (NHANES), 1999 to 2018.

## References

[1] Petersen M.C., Shulman G.I. (2018). Mechanisms of Insulin Action and Insulin Resistance. Physiol. Rev..

[2] Shanik M.H., Xu Y., Skrha J., Dankner R., Zick Y., Roth J. (2008). Insulin resistance and hyperinsulinemia: Is hyperinsulinemia the cart or the horse?. Diabetes Care.

[3] Corkey B.E. (2012). Banting lecture 2011: Hyperinsulinemia: Cause or consequence?. Diabetes.

[4] Nolan C.J., Prentki M. (2019). Insulin resistance and insulin hypersecretion in the metabolic syndrome and type 2 diabetes: Time for a conceptual framework shift. Diabetes Vasc. Dis. Res..

[5] Nolan C.J., Ruderman N.B., Kahn S.E., Pedersen O., Prentki M. (2015). Insulin resistance as a physiological defense against metabolic stress: Implications for the management of subsets of type 2 diabetes. Diabetes.

[6] Abdul-Ghani M., DeFronzo R.A. (2021). Insulin Resistance and Hyperinsulinemia: The Egg and the Chicken. J. Clin. Endocrinol. Metab..

[7] Gluvic Z., Zaric B., Resanovic I., Obradovic M., Mitrovic A., Radak D., Isenovic E. (2017). Link between Metabolic Syndrome and Insulin Resistance. Curr. Vasc. Pharmacol..

[8] Thomas D.D., Corkey B.E., Istfan N.W., Apovian C.M. (2019). Hyperinsulinemia: An Early Indicator of Metabolic Dysfunction. J. Endocr. Soc..

[9] Janssen J. (2021). Hyperinsulinemia and Its Pivotal Role in Aging, Obesity, Type 2 Diabetes, Cardiovascular Disease and Cancer. Int. J. Mol. Sci..

[10] Kosmas C.E., Bousvarou M.D., Kostara C.E., Papakonstantinou E.J., Salamou E., Guzman E. (2023). Insulin resistance and cardiovascular disease. J. Int. Med. Res..

[11] Arcidiacono B., Iiritano S., Nocera A., Possidente K., Nevolo M.T., Ventura V., Foti D., Chiefari E., Brunetti A. (2012). Insulin Resistance and Cancer Risk: An Overview of the Pathogenetic Mechanisms. Exp. Diabetes Res..

[12] Tanase D.M., Gosav E.M., Costea C.F., Ciocoiu M., Lacatusu C.M., Maranduca M.A., Ouatu A., Floria M. (2020). The Intricate Relationship between Type 2 Diabetes Mellitus (T2DM), Insulin Resistance (IR), and Nonalcoholic Fatty Liver Disease (NAFLD). J. Diabetes Res..

[13] Dunaif A. (1997). Insulin Resistance and the Polycystic Ovary Syndrome: Mechanism and Implications for Pathogenesis. Endocr. Rev..

[14] Sarafidis P.A. (2008). Obesity, insulin resistance and kidney disease risk: Insights into the relationship. Curr. Opin. Nephrol. Hypertens..

[15] Fragoso A., Mendes F., Silva A.P., Neves P.L. (2015). Insulin resistance as a predictor of cardiovascular morbidity and end-stage renal disease. J. Diabetes Complicat..

[16] Sędzikowska A., Szablewski L. (2021). Insulin and Insulin Resistance in Alzheimer’s Disease. Int. J. Mol. Sci..

[17] Gulli G., Ferrannini E., Stern M., Haffner S., Defronzo R.A. The Metabolic Profile of NIDDM Is Fully Established in Glucose-Tolerant Offspring of Two Mexican-American NIDDM Parents. http://diabetesjournals.org/diabetes/article-pdf/41/12/1575/359351/41-12-1575.pdf.

[18] Moore J.X., Chaudhary N., Akinyemiju T. (2017). Metabolic Syndrome Prevalence by Race/Ethnicity and Sex in the United States, National Health and Nutrition Examination Survey, 1988–2012. Prev. Chronic Dis..

[19] Hirode G., Wong R.J. (2020). Trends in the Prevalence of Metabolic Syndrome in the United States, 2011–2016. JAMA.

[20] Menke A., Casagrande S., Geiss L., Cowie C.C. (2015). Prevalence of and Trends in Diabetes Among Adults in the United States, 1988-2012. JAMA.

[21] Wang L., Li X., Wang Z., Bancks M.P., Carnethon M.R., Greenland P., Feng Y.-Q., Wang H., Zhong V.W. (2021). Trends in Prevalence of Diabetes and Control of Risk Factors in Diabetes Among US Adults, 1999–2018. JAMA.

[22] He J., Zhu Z., Bundy J.D., Dorans K.S., Chen J., Hamm L.L. (2021). Trends in Cardiovascular Risk Factors in US Adults by Race and Ethnicity and Socioeconomic Status, 1999–2018. JAMA.

[23] Cowie C.C., Casagrande S.S., Menke A., Cissell M.A., Eberhardt M.S., Meigs J.B., Gregg E.W., Knowler W.C., Barrett-Connor E., Becker D.J. (2018). Diabetes in America.

[24] Li C., Ford E.S., McGuire L.C., Mokdad A.H., Little R.R., Reaven G.M. (2006). Trends in hyperinsulinemia among nondiabetic adults in the U.S. Diabetes Care.

[25] Mokdad A.H., Ballestros K., Echko M., Glenn S., Olsen H.E., Mullany E., Lee A., Khan A.R., Ahmadi A., US Burden of Disease Collaborators (2018). The state of US health, 1990–2016: Burden of diseases, injuries, and risk factors among US states. JAMA.

[26] Zipf G., Chiappa M., Porter K.S., Ostchega Y., Lewis B.G., Dostal J. (2013). National health and nutrition examination survey: Plan and operations, 1999–2010. Vital Health Stat. 1.

[27] US Department of Health & Human Services Poverty Guidelines, Research, and Measurement.

[28] Ogden C.L., Carroll M.D., Fakhouri T.H., Hales C.M., Fryar C.D., Li X., Freedman D.S. (2018). Morbidity and Mortality Weekly Report Prevalence of Obesity Among Youths by Household Income and Education Level of Head of Household-United States 2011–2014. MMWR Morb. Mortal. Wkly. Rep..

[29] NHANES 2003–2004: Plasma Fasting Glucose, Serum C-peptide & Insulin Data Documentation, Codebook, and Frequencies. https://wwwn.cdc.gov/Nchs/Data/Nhanes/Public/2003/DataFiles/L10AM_C.htm.

[30] NHANES 2005–2006: Plasma Fasting Glucose & Insulin Data Documentation, Codebook, and Frequencies. https://wwwn.cdc.gov/Nchs/Data/Nhanes/Public/2005/DataFiles/GLU_D.htm.

[31] NHANES 2011–2012: Plasma Fasting Glucose & Insulin Data Documentation, Codebook, and Frequencies. https://wwwn.cdc.gov/Nchs/Data/Nhanes/Public/2011/DataFiles/GLU_G.htm.

[32] INS_H. https://wwwn.cdc.gov/Nchs/Data/Nhanes/Public/2013/DataFiles/INS_H.htm.

[33] Matthews D.R., Hosker J.P., Rudenski A.S., Naylor B.A., Treacher D.F., Turner R.C. (1985). Homeostasis model assessment: Insulin resistance and beta-cell function from fasting plasma glucose and insulin concentrations in man. Diabetologia.

[34] Bonora E., Kiechl S., Willeit J., Oberhollenzer F., Egger G., Targher G., Alberiche M., Bonadonna R.C., Muggeo M. (1998). Prevalence of insulin resistance in metabolic disorders: The Bruneck Study. Diabetes.

[35] Emoto M., Nishizawa Y., Maekawa K., Hiura Y., Kanda H., Kawagishi T., Shoji T., Okuno Y., Morii H. (1999). Homeostasis model assessment as a clinical index of insulin resistance in type 2 diabetic patients treated with sulfonylureas. Diabetes Care.

[36] Tahapary D.L., Pratisthita L.B., Fitri N.A., Marcella C., Wafa S., Kurniawan F., Rizka A., Tarigan T.J.E., Harbuwono D.S., Purnamasari D. (2022). Challenges in the diagnosis of insulin resistance: Focusing on the role of HOMA-IR and Tryglyceride/glucose index. Diabetes Metab. Syndr..

[37] Gastaldelli A. (2022). Measuring and estimating insulin resistance in clinical and research settings. Obesity.

[38] Sumner A.E., Cowie C.C. (2008). Ethnic differences in the ability of triglyceride levels to identify insulin resistance. Atherosclerosis.

[39] Johnson C.L., Dohrmann S.M., Burt V.L., Mohadjer L.K. (2014). National health and nutrition examination survey: Sample design, 2011–2014. Vital Health Stat. 2.

[40] Curtin L.R., Mohadjer L.K., Dohrmann S.M., Montaquila J.M., Kruszan-Moran D., Mirel L.B., Carroll M.D., Hirsch R., Schober S., Johnson C.L. (2012). The National Health and Nutrition Examination Survey: Sample Design, 1999–2006. Vital Health Stat. 2.

[41] Curtin L.R., Mohadjer L.K., Dohrmann S.M., Kruszon-Moran D., Mirel L.B., Carroll M.D., Hirsch R., Burt V.L., Johnson C.L. (2013). National Health and Nutrition Examination Survey: Sample design, 2007–2010. Vital Health Stat. 2.

[42] Chen T.C., Clark J., Riddles M.K., Mohadjer L.K., Fakhouri T.H.I. (2020). National Health and Nutrition Examination Survey, 2015–2018: Sample Design and Estimation Procedures. Vital Health Stat. 2.

[43] Age and Sex Composition in the United States: 2010. https://www.census.gov/data/tables/2010/demo/age-and-sex/2010-age-sex-composition.html.

[44] Ingram D.D., Malec D.J., Makuc D.M., Kruszon-Moran D., Gindi R.M., Albert M., Beresovsky V., Hamilton B.E., Holmes J., Schiller J. (2018). National Center for Health Statistics Guidelines for Analysis of Trends. Vital Health Stat. 2.

[45] Joinpoint Regression Program. https://surveillance.cancer.gov/joinpoint/.

[46] Kim H.J., Fay M.P., Yu B., Barrett M.J., Feuer E.J. (2004). Comparability of segmented line regression models. Biometrics.

[47] Du Y., Liu B., Sun Y., Snetselaar L.G., Wallace R.B., Bao W. (2019). Trends in Adherence to the Physical Activity Guidelines for Americans for Aerobic Activity and Time Spent on Sedentary Behavior among US Adults, 2007 to 2016. JAMA Netw Open..

[48] Ciarambino T., Crispino P., Guarisco G., Giordano M. (2023). Gender Differences in Insulin Resistance: New Knowledge and Perspectives. Curr. Issues Mol. Biol..

[49] Gado M., Tsaousidou E., Bornstein S.R., Perakakis N. (2024). Sex-based differences in insulin resistance. J. Endocrinol..

[50] Hasson B.R., Apovian C., Istfan N. (2015). Racial/Ethnic Differences in Insulin Resistance and Beta Cell Function: Relationship to Racial Disparities in Type 2 Diabetes among African Americans versus Caucasians. Curr. Obes. Rep..

[51] Armiyaw L., Sarcone C., Fosam A., Muniyappa R. (2020). Increased β-cell responsivity independent of insulin sensitivity in healthy African American adults. J. Clin. Endocrinol. Metab..

[52] Rehm C.D., Peñalvo J.L., Afshin A., Mozaffarian D. (2016). Dietary intake among US Adults, 1999–2012. JAMA.

[53] Fuller-Rowell T.E., Homandberg L.K., Curtis D.S., Tsenkova V.K., Williams D.R., Ryff C.D. (2019). Disparities in insulin resistance between black and white adults in the United States: The role of lifespan stress exposure. Psychoneuroendocrinology.

[54] Fakhouri T.H.I., Martin C.B., Chen T.C., Akinbami L.J., Ogden C.L., Paulose-Ram R., Riddles M.K., Van de Kerckhove W., Roth S.B., Clark J. (2020). An Investigation of Nonresponse Bias and Survey Location Variability in the 2017–2018 National Health and Nutrition Examination Survey. Vital Health Stat. 2.

[55] American Diabetes Association Professional Practice Committee (2021). 2. Classification and Diagnosis of Diabetes: Standards of Medical Care in Diabetes—2022. Diabetes Care.

