# Supplementary material for: Trends in Hyperinsulinemia and Insulin Resistance Among Nondiabetic US Adults, NHANES, 1999–2018
Source: J Clin Med. 2025 May 6;14(9):3215. doi: 10.3390/jcm14093215 (PMC12072812; doi:10.3390/jcm14093215)
Supplement: Supplementary file 1 [file jcm-14-03215-s001.zip › jcm-3564884-supplementary.pdf]

Supplementary

Trends in Hyperinsulinemia and Insulin Resistance Among Nondiabetic US Adults, NHANES, 1999–2018

Chuyue Wu, Yixun Ke, Roch A. Nianogo

Section. Trends in the means of natural log-scale fasting insulin levels and natural log-scale HOMA-IR index levels by sociodemographic subgroups

Table S1. The Change of Insulin Measurement Method and Recommended Adjustment Formula from the NHANES Website.
Table S2. Test for Trend in the Means of Log-scale Fasting Insulin Levels Stratified by Sex, Race/Ethnicity, Educational and Income level, National Health and Nutrition Examination Survey (NHANES), 1999 to 2018, n=17,310
Table S3. Test for Trend in the Means of Log-scale HOMA-IR Index Stratified by Sex, Race/Ethnicity, Educational and Income level, National Health and Nutrition Examination Survey (NHANES), 1999 to 2018, n=17,310

Figure S1. Age-standardized Mean and 95%CI of Natural Log-scale Fasting Insulin Levels Stratified by Sex, Race/Ethnicity, Educational and Income level, National Health and Nutrition Examination Survey (NHANES), 1999 to 2018
Figure S2. Age-standardized Mean and 95%CI of Natural Log-scale HOMA-IR Index Stratified by Sex, Race/Ethnicity, Educational and Income level, National Health and Nutrition Examination Survey (NHANES), 1999 to 2018

Section.

Trends in the means of natural log-scale fasting insulin levels by sociodemographic subgroups

eTable 2 and eFigure 1 illustrated the means of log scale fasting insulin levels and the trends. In the total population, age-standardized means of natural log fasting insulin levels increased from 1.9 (95%CI: 1.8-2.0) in 1999-2000 to 2.2 (95%CI: 2.1-2.2) in 2017-2018 with an increasing linear trend (Slope=0.036, SE=0.004, p for trend <0.0001). A nonlinear trend was observed for female participants, with the insulin levels remaining stable from 1999 to 2005, followed by a rapidly increasing trend from 2005 to 2018 (Slope=0.055, SE=0.008, p for trend <0.0001). Male people had insulin levels generally higher than females throughout the periods, but the differences between sexes were insignificant. We observed positive linear trends across all racial/ethnic subgroups. The Asian population had the fastest increasing rate compared to other racial/ethnic people. Hispanic and non-Hispanic Black populations had relatively higher levels of age-standardized means of natural log fasting insulin when compared to non-Hispanic White and Asian populations (eFigure 2). No significant disparities were detected within the various subgroups based on education or family income levels. The trends did not change much in the fully adjusted models.

Trends in the means of natural log-scale HOMA-IR index levels by sociodemographic subgroups

eTable 3 and eFigure 2 revealed a consistent upward trend in the log scale HOMA-IR index levels from 1999 to 2018, mirroring the patterns observed for fasting insulin levels. This rise was reflected across all subgroups, with a particularly notable increase trend among non-Hispanic Asian individuals (slope=0.094, SE=0.026, p for trend 0.0006) since 2011. After adjusting for sex, education, and poverty level, non-Hispanic Black individuals experienced the slowest increase trend (slope=0.025, SE=0.006, p for trend < 0.0001) from 1999 to 2018 compared to other racial/ethnic groups. Both male and female populations showed an increased trend, while females have a steeper incline than males (slope 0.050 vs. 0.035, interaction p-value: 0.015). Individuals from all educational and income levels showed a positive trend in the HOMA-IR index, with no significant difference detected across subgroups.

Table S1. The Change of Insulin Measurement Method and Recommended Adjustment Formula from the NHANES website.

Cycle	Location	Method	Adjustments for insulin values across laboratory methods[29–32]
1999-2000	University of Missouri-Columbia	Pharmacia Analyzer Radioimmunoassay (RIA)	Tosoh-equivalent Insulin = 1.0027 * Pharmacia Insulin - 2.2934
2001-2002	University of Missouri-Columbia	Pharmacia Analyzer Radioimmunoassay (RIA)	Tosoh-equivalent Insulin = 1.0027 * Pharmacia Insulin - 2.2934
2003-2004	University of Missouri-Columbia	Tosoh Analyzer Immunoenzymometric Assay	NA
2005-2006	University of Minnesota	Mercodia Analyzer Immunoenzymometric Assay	Tosoh-equivalent Insulin = 1.0526 * Mercodia Insulin – 1.5674
2007-2008	University of Minnesota	Mercodia Analyzer Immunoenzymometric Assay	Tosoh-equivalent Insulin = 1.0526 * Mercodia Insulin – 1.5674
2009-2010	University of Minnesota	2009: Mercodia ELISA Human Insulin Immunoassay<br><br>2010: Human Insulin Immunoassay Using ROCHE ELECSYS	Roche-equivalent Insulin = 0.8868 * Mercodia Insulin + [0.0011 * (Mercodia Insulin) ^ 2] – 0.0744<br><br>Tosoh-equivalent Insulin = 10 ^ [1.024 * log10(Roche insulin) – 0.0802]
2011-2012	University of Minnesota	Roche Analyzer Chemiluminescent "sandwich" immunoassay	Tosoh-equivalent Insulin = 10 ^ (1.024 * log10(Roche insulin) – 0.0802)
2013-2014	University of Missouri-Columbia	Tosoh AIA-900 Chemistry Analyzer Immunoenzymometric Assay	NA
2015-2016	University of Missouri-Columbia	Tosoh AIA-900 Chemistry Analyzer Immunoenzymometric Assay	NA
2017-2018	University of Missouri-Columbia	Tosoh AIA-900 Chemistry Analyzer Immunoenzymometric Assay	NA

Table S2. Test for Trend in the Means of Log-scale Fasting Insulin Levels Stratified by Sex, Race/Ethnicity, Educational and Income level, National Health and Nutrition Examination Survey (NHANES), 1999 to 2018, n=17,310

	Age-adjusted models				
	**Joinpoint wave†**	**slope (SE) p-value** Segment 1	Segment 2	**Contrast p-value‡**	**Interaction p-value§**
**Overall**	NA	0.036 (0.004) <0.001		NA	NA
**Sex**					
Female	4	0.010 (0.016) 0.51	0.055 (0.008) <0.001	0.046	0.014
Male	NA	0.029 (0.005) <0.001		NA	ref
**Race/Ethnicity‖**					
Non-Hispanic White	NA	0.036 (0.005) <0.001		NA	ref
Non-Hispanic Black	NA	0.023 (0.005) <0.001		NA	0.078

Hispanic	NA	0.033 (0.006)<br><0.001		NA	0.62
Non-Hispanic Asian[¶]	NA	0.068 (0.022)<br>0.003		NA	0.013
**Educational level[**]**					
High school or less	NA	0.036 (0.005)<br><0.001		NA	0.72
Some college or associate's degree	8	0.055 (0.008)<br><0.001	-0.037 (0.033)<br>0.26	0.015	0.80
College graduate or higher	NA	0.039 (0.006)<br><0.001		NA	ref
**Poverty-Income Ratio[††]**					
Ratio<=1.3	NA	0.031 (0.006)<br><0.001		NA	0.98
1.3<Ratio<=3.5	NA	0.040 (0.006)<br><0.001		NA	0.21
Ratio>3.5	NA	0.031 (0.006)<br><0.001		NA	ref

	**Fully-adjusted[‡‡] models**				
	**Joinpoint wave[†]**	**slope (SE) p-value**		**Contrast p-value[‡]**	**Interaction p-value[§]**
		Segment 1	Segment 2		
**Overall**	NA	0.035 (0.004)<br><0.001		NA	NA
**Sex**					
Female	NA	0.042 (0.005)<br><0.001		NA	0.003
Male	NA	0.029 (0.005)<br><0.001		NA	ref
**Race/Ethnicity[‖]**					
Non-Hispanic White	NA	0.038 (0.005)<br><0.001		NA	ref
Non-Hispanic Black	NA	0.019 (0.006)<br>0.001		NA	0.010
Hispanic	NA	0.034 (0.006)<br><0.001		NA	0.65
Non-Hispanic Asian[¶]	NA	0.056 (0.024)<br>0.021		NA	0.037
**Educational level[**]**					
High school or less	NA	0.032 (0.005)<br><0.001		NA	0.63
Some college or associate's degree	8	0.054 (0.009)<br><0.001	-0.034 (0.034)<br>0.33	0.027	0.63
College graduate or higher	NA	0.036 (0.007)<br><0.001		NA	ref
**Poverty-Income Ratio[††]**					
Ratio<=1.3	NA	0.031 (0.006)<br><0.001		NA	0.78
1.3<Ratio<=3.5	NA	0.040 (0.006)<br><0.001		NA	0.25
Ratio>3.5	NA	0.034 (0.005)<br><0.001		NA	ref

Abbreviations: SE, standard error; ref, reference; NA, not applicable.

† Nonlinearity was assessed by testing for the statistical significance of the cubic term and quadratic term of survey cycles in the polynomial logistic regression models. The locations of joinpoint waves were identified by using the NCI's Joinpoint software for the nonlinear trends.
‡ Contrast p-value tested for the statistical significance of the difference between two segments.
§ Interaction p-value tested for the statistical significance of the interaction term between the potential modifiers and survey cycle.
|| In the race/ethnicity subgroup analyses, race/ethnicity was categorized as non-Hispanic White, non-Hispanic Black, Hispanic, Other. All ten survey cycles are included with sample size 17,310.
¶ In the non-Hispanic Asian subgroup analyses, since representative information for non-Hispanic Asian population was available in the NHANES only from 2011 through 2018, the analytic sample size was 7,066 (i.e., the study population from last for survey cycles, 2011 to 2018).
** Educational level had 19 missing values which were excluded from the analyses related to educational level.
†† Poverty-Income Ratio had 1,538 missing values which were excluded from the analyses related to Poverty-Income Ratio.
‡‡ The fully-adjusted models were adjusted for age, sex, race/ethnicity (non-Hispanic White, non-Hispanic Black, Hispanic, Other), educational level, and poverty-income ratio. In the subgroup analyses, the stratified variable was eliminated from the fully-adjusted models, correspondingly.

Table S3. Test for Trend in the Means of Log-scale HOMA-IR Index Stratified by Sex, Race/Ethnicity, Educational and Income level, National Health and Nutrition Examination Survey (NHANES), 1999 to 2018, n=17,310

	Age-adjusted models				
	**Joinpoint wave†**	**slope (SE) p-value**		**Contrast p-value‡**	**Interaction p-value§**
		Segment 1	Segment 2		
**Overall**	NA	0.044 (0.004) <0.001		NA	NA
**Sex**					
Female	NA	0.050 (0.005) <0.001		NA	0.015
Male	NA	0.035 (0.005) <0.001		NA	Ref
**Race/Ethnicity||**					
Non-Hispanic White	NA	0.043 (0.005) <0.001		NA	Ref
Non-Hispanic Black	NA	0.029 (0.006) <0.001		NA	0.083
Hispanic	NA	0.039 (0.006) <0.001		NA	0.65
Non-Hispanic Asian¶	NA	0.094 (0.026) <0.001		NA	0.013
**Educational level****					
High school or less	NA	0.042 (0.005) <0.001		NA	0.75
Some college or associate's degree	8	0.061 (0.009) <0.001	-0.024 (0.034) 0.48	0.032	0.75
College graduate or higher	NA	0.045 (0.007) <0.001		NA	Ref
**Poverty-Income Ratio††**					
Ratio<=1.3	NA	0.036 (0.006) <0.001		NA	0.93
1.3<Ratio<=3.5	NA	0.047 (0.006) <0.001		NA	0.193

Ratio>3.5	NA	0.037 (0.006)<br><0.001		NA	Ref

Fully-adjusted[‡‡] models					
	**Joinpoint wave[†]**	**slope (SE) p-value**		**Contrast p-value[‡]**	**Interaction p-value[§]**
		Segment 1	Segment 2		
**Overall**	NA	0.042 (0.004)<br><0.001		NA	NA
**Sex**					
Female	NA	0.049 (0.005)<br><0.001		NA	0.013
Male	NA	0.035 (0.005)<br><0.001		NA	Ref
**Race/Ethnicity[||]**					
Non-Hispanic White	NA	0.045 (0.005)<br><0.001		NA	Ref
Non-Hispanic Black	NA	0.025 (0.006)<br><0.001		NA	0.013
Hispanic	NA	0.041 (0.006)<br><0.001		NA	0.69
Non-Hispanic Asian[¶]	NA	0.076 (0.025)<br>0.003		NA	0.029
**Educational level[**]**					
High school or less	NA	0.038 (0.005)<br><0.001		NA	0.63
Some college or associate's degree	8	0.060 (0.009)<br><0.001	-0.021 (0.036)<br>0.56	0.050	0.60
College graduate or higher	NA	0.042 (0.007)<br><0.001		NA	Ref
**Poverty-Income Ratio[††]**					
Ratio<=1.3	NA	0.036 (0.006)<br><0.001		NA	0.68
1.3<Ratio<=3.5	NA	0.048 (0.006)<br><0.001		NA	0.24
Ratio>3.5	NA	0.041 (0.006)<br><0.001		NA	Ref

Abbreviations: SE, standard error; ref, reference; NA, not applicable.

[†] Nonlinearity was assessed by testing for the statistical significance of the cubic term and quadratic term of survey cycles in the polynomial logistic regression models. The locations of joinpoint waves were identified by using the NCI's Joinpoint software for the nonlinear trends.

[‡] Contrast p-value tested for the statistical significance of the difference between two segments.

[§] Interaction p-value tested for the statistical significance of the interaction term between the potential modifiers and survey cycle.

[||] In the race/ethnicity subgroup analyses, race/ethnicity was categorized as non-Hispanic White, non-Hispanic Black, Hispanic, Other. All ten survey cycles are included with sample size 17,310.

[¶] In the non-Hispanic Asian subgroup analyses, since representative information for non-Hispanic Asian population was available in the NHANES only from 2011 through 2018, the analytic sample size was 7,066 (i.e., the study population from last 4 survey cycles, 2011 to 2018).

[**] Educational level had 19 missing values which were excluded from the analyses related to educational level.

[††] Poverty-Income Ratio had 1,538 missing values which were excluded from the analyses related to Poverty-Income Ratio.

[‡‡] The fully-adjusted models were adjusted for age, sex, race/ethnicity (non-Hispanic White, non-Hispanic Black, Hispanic, Other), educational level, and poverty-income ratio. In the subgroup analyses, the stratified variable was eliminated from the fully-adjusted models, correspondingly.

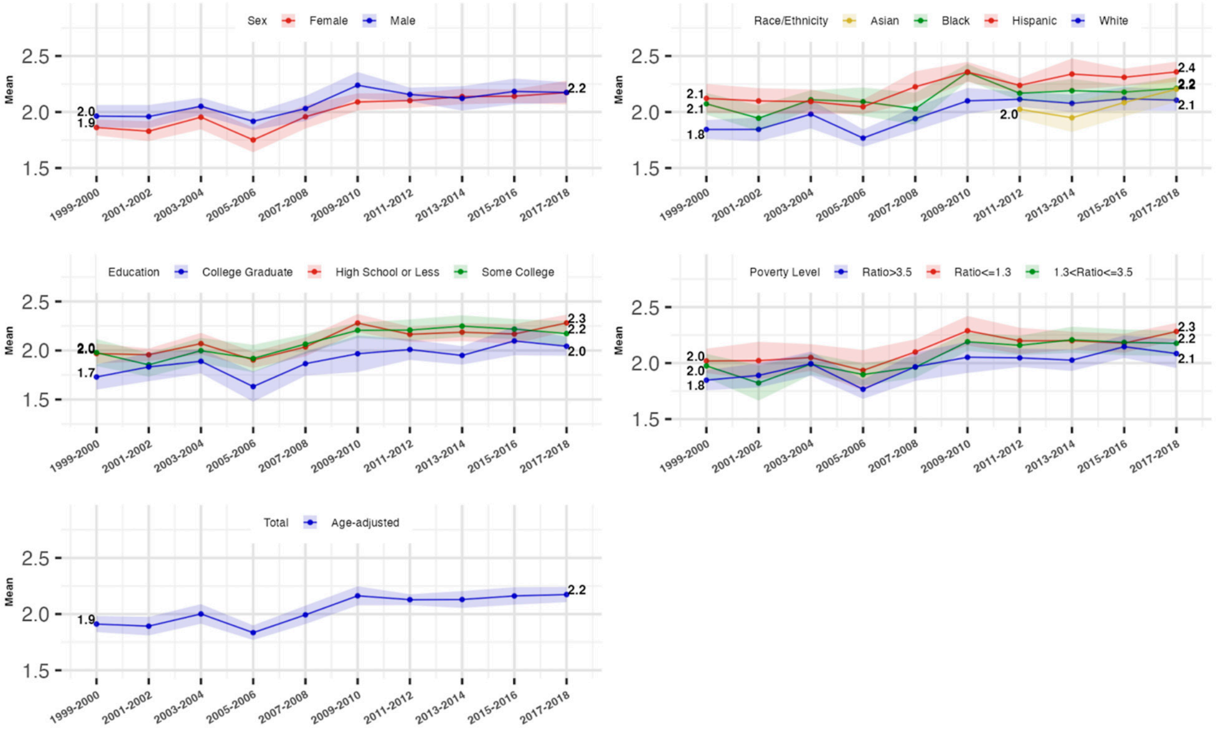

Figure S1. *Age-standardized Mean and 95% Confidence Interval of Natural Log-scale Fasting Insulin Levels Stratified by Sex, Race/Ethnicity, Educational and Income level, National Health and Nutrition Examination Survey (NHANES), 1999 to 2018*

Figure legend: Showing the trends in the mean of natural log-scale fasting insulin level and the disparities across sociodemographic groups. The mean of natural log-scale fasting insulin level and the corresponding 95% confidence interval were survey sample weighted and age-standardized to 2010 U.S. Census adult population. The sample size for the total, sex-stratified, and race/ethnicity (excluding non-Hispanic Asians)-stratified trends was 17,310. The sample size for the stratified non-Hispanic Asian subgroup was 7,066. 19 participants were excluded from the education-stratified trend analyses. 1538 participants were excluded from the income-stratified trend analyses.

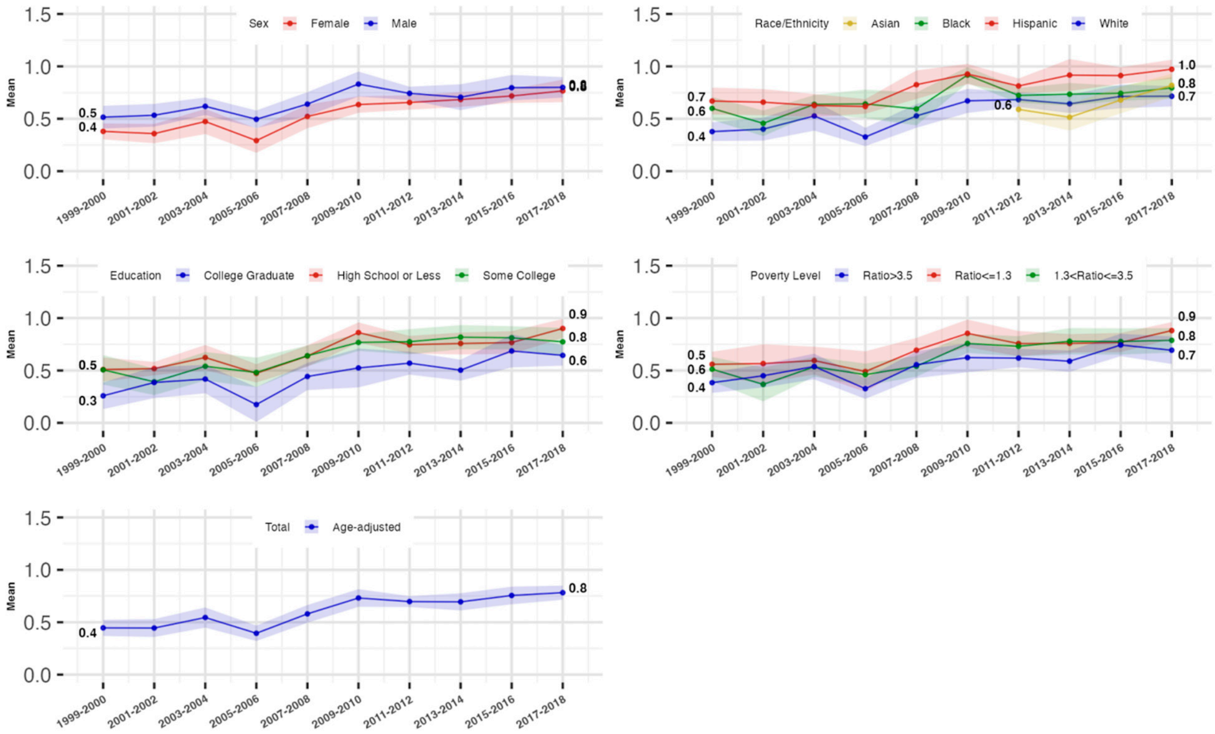

Figure S2*. Age-standardized Mean and 95% Confidence Interval of Natural Log-scale HOMA-IR Index Stratified by Sex, Race/Ethnicity, Educational and Income level, National Health and Nutrition Examination Survey (NHANES), 1999 to 2018*

Figure legend: Showing the trends in the mean of natural log-scale HOMA-IR index and the disparities across sociodemographic groups. The mean of natural log-scale HOMA-IR index and the corresponding 95% confidence interval were survey sample weighted and age-standardized to 2010 U.S. Census adult population. The sample size for the total, sex-stratified, and race/ethnicity (excluding non-Hispanic Asian)-stratified trends was 17,310. The sample size for the stratified non-Hispanic Asian subgroup was 7,066. 19 participants were excluded from the education-stratified trend analyses. 1538 participants were excluded from the income-stratified trend analyses. Abbreviation: HOMA-IR, Homeostatic Model Assessment of Insulin Resistance.
